# Preference, acceptability and implications of the rapid hepatitis C screening test among high-risk young people who inject drugs

**DOI:** 10.1186/1471-2458-14-645

**Published:** 2014-06-25

**Authors:** Benjamin Hayes, Alya Briceno, Alice Asher, Michelle Yu, Jennifer L Evans, Judith A Hahn, Kimberly Page

**Affiliations:** 1 Department of Epidemiology and Biostatistics and Global Health Science, University of California San Francisco, 50 Beale Street, Suite 1200, San Francisco, CA 94105, USA; 2 Department of Community Health Systems, School of Nursing, University of California San Francisco, 505 Parnassus Ave, San Francisco, CA 94122, USA; 3 Department of Medicine, San Francisco General Hospital, University of California San Francisco, 1001 Potrero Ave, San Francisco, CA 94110, USA

**Keywords:** Hepatitis C virus, Rapid testing, Injection drug users

## Abstract

**Background:**

People who inject drugs (PWID) are at highest risk for hepatitis C virus (HCV) infection, yet many remain unaware of their infection status. New anti-HCV rapid testing has high potential to impact this.

**Methods:**

Young adult (<30 years) active PWID were offered either the rapid OraQuick® or standard anti-HCV test involving phlebotomy, then asked to complete a short questionnaire about testing perceptions and preferences. Sample characteristics, service utilization, and injection risk exposures are assessed with the HCV testing choice as the outcome, testing preferences, and reasons for preference.

**Results:**

Of 129 participants: 82.9% (n = 107) chose the rapid test. There were no significant differences between those who chose rapid vs. standard testing. A majority (60.2%) chose the rapid test for quick results; most (60.9%) felt the rapid test was accurate, and less painful (53.3%) than the tests involving venipuncture.

**Conclusions:**

OraQuick® anti-HCV rapid test was widely accepted among young PWID. Our results substantiate the valuable potential of anti-HCV rapid testing for HCV screening in this high risk population.

## Background

Hepatitis C virus (HCV) remains a serious public health problem [1]. In the US, National Health and Nutrition Examination Surveys (NHANES) data estimate 2.2-3.2 million people in the U.S. are living with HCV, which is likely an underestimate [2]. Previous analysis has estimated that NHANES does not account for an additional 1.1 million infected persons [3,4]. HCV is a significant cause of liver disease, cirrhosis, and liver cancer [5,6], and now surpasses HIV as a cause of death [7]. Injection drug use is the leading behavioral risk factor for HCV infection [2,8] and 75%-90% of long-term people who inject drugs (PWID) are reported to be anti-HCV positive [9].

Despite overall declining incidence of HCV infection in the US since 1992 [8], recent outbreaks of HCV have been documented among young adult PWID [10-13]. These trends coincide with increasing rates of prescription opiate misuse among youth, which may confer higher risks of HCV transmission [14,15]. Young PWID also contend with the exceptional rates of seroconversion associated with the first few years of initiating injection drug use [16] and are often reliant upon social relationships in which risk-related injection behaviors, including sharing non-sterile injection equipment, are conducted [17,18].

The U.S. Centers for Disease Control and Prevention and U.S. Prevention Services (CDC) Task Force recommends routine anti-HCV testing for anyone who has injected drugs and one-time testing for adults born between 1945–1965 [19,20]. Testing for HCV may enhance prevention efforts, improve efforts to identify people eligible for treatment, and increase uptake into care [3,21-24]. Some studies suggest that PWID who know their serostatus may reduce some high-risk injection-related behaviors [25-27], however others have not observed this [22,24,28]. Young PWID may also engage in seroadaptive practices, based on their perception of their partners’ HCV status [18,29], although others have not observed this [22,24,28,30,31]. Finally, identifying HCV infections among PWID is critical for ensuring referrals to appropriate care and treatment.

Many of those at risk for and living with HCV are unaware of their serostatus [3,26]. One multi-city study reported that as many as 72% of anti-HCV positive young injectors did not know their status [25]. The low numbers of PWID who test for HCV may be due in small or large part to the requirements of standard testing algorithms, which include at the least, an enzyme immunoassay (EIA) followed by an HCV RNA test. These tests, which involve phlebotomy and laboratory work, significant waiting times, as well as potentially re-drawing of blood (for RNA confirmatory testing) are potential barriers to testing and results disclosure for people, including PWID, who may be homeless, lack access to health insurance, unable or unwilling to return for results, or not have accessible veins [21,32,33].

Treatment of HCV has been previously shown to be feasible and just as effective among PWID as non-injectors [34-37], yet many PWID have avoided treatment due to fears of the adverse effects of the medications and the difficulties of balancing life circumstances and comorbidities, or have been restricted by physicians who anticipate poor adherence [37]. Advances in HCV treatment options improve side-effect profiles, potentially shorten the course of treatment and increase rates of sustained virologic response (SVR) [38]. These advances may increase demand and access for care and treatment among young adult PWID, who historically have had little access to HCV treatment [39,40], as well as enhance the incentive to know one’s HCV status, simultaneously increasing the value of faster, easier and more accessible testing options.

Rapid HIV testing has been demonstrated to be acceptable among young, high-risk and traditionally hard-to-reach populations and in community-based venues [41-44] and has been associated with an increased test and disclosure acceptance [43,45] and entering care [46]. Recently available anti-HCV rapid testing is similarly accurate to conventional EIA testing [47,48] and has been demonstrated to be appropriate in community settings [21]. Comparisons between the HIV and HCV tests are limited, however by the potentially different meanings of each test (positive anti-HIV antibody is either a) positive viral infection, or b) false positive, whereas a positive anti-HCV test may indicate either a) positive viral infection, b) past, resolved infection, or c) false positive). Despite these differences, it seems reasonable to assume that similar levels of preference and acceptability toward the rapid HCV may also extrapolate to increased number of people who test for HCV, know their HCV status and enter care.

To date, we know of no studies that analyze testing preference and acceptability of anti-HCV rapid tests against traditional phlebotomy-based blood draw testing among young adult PWID. This study of participants of a prospective observational cohort of young PWID measures 1) choice of either rapid testing finger prick or standard laboratory-based testing via venipuncture and 2) levels of preference and acceptability toward the rapid test based on a short questionnaire. Preliminary results showed high levels of preference and acceptability for the rapid test [49].

## Methods

### Study population data collection

The UFO Study is an ongoing prospective observational study of HCV risk and incidence in young PWID; results and methods have been described in detail elsewhere [17,50]. For this sub-study, newly enrolled participants who completed a baseline visit (Visit 1 of the prospective study) between June 2012 and August 2013 were approached to complete the HCV rapid test acceptability survey. Participants enrolled prior to June 2012 were approached at a scheduled quarterly follow-up study visit. Baseline visit eligibility criteria included subjects under 30 years of age, self-report having injected in the last 30 days at baseline, and self-report negative or unknown HCV RNA status; prospective participants with unknown HCV status, or anti-HCV positive but HCV RNA negative, were eligible to participate in the study. Baseline and follow-up visits included both anti-HCV testing and HCV RNA testing, as well as completion of an interviewer-administered behavioral survey. Effective June 2012, participants were offered a choice between anti-HCV rapid testing (OraSure© Technologies: Bethlehem, PA) and standard laboratory-based anti-HCV testing via venipuncture beginning in June 2012. When study participants chose the rapid test method, results were available and disclosed at that time, and one week later for those who chose phlebotomy-based testing. Venipuncture for HCV RNA testing was routine for these study visits (for HCV viral RNA testing) as well as collection of dried blood spot (DBS) [51], therefore all participants underwent both phlebotomy and finger prick testing regardless of test choice. HCV rapid test results were disclosed prior to the administration of the HCV rapid test survey. All participants received pre- and post-test risk reduction counseling. Health care referrals were provided for participants with HCV infection. The protocol for this study was reviewed and approved by the UCSF Human Research Protection Institutional Review Board. Written informed consent was obtained from all participants.

Data and specimen collection occurred at UCSF Clinical Research Services’ Tenderloin Clinical Research Center (TCRC), which has a Clinical Laboratory Improvement Amendments (CLIA) waiver, a regulatory institution overseeing laboratory testing of humans in the US that waives certification for laboratories that perform simple tests as determined by the Food and Drug Administration (FDA) and Center for Disease Control and Prevention (CDC). The research staff included trained phlebotomists, interviewers, and counselors who had training and experience with HIV and HCV testing protocols and risk reduction counseling with the PWID population.

### Measures

Data collected from the behavioral survey, including: (a) participant sociodemographic characteristics, (b) previous HIV testing history and self-reported anti-HIV status, (c) service utilization, including having used needle exchange in the past 3 months; and (d) risk behaviors associated with number of years injecting was assessed in association with anti-HCV testing preference (rapid vs. standard).

The ‘rapid HCV test survey’ measured preferences, perceptions and reasons for testing choice. This survey, adapted from a previously published survey assessing acceptability of rapid HIV testing [52], asked ‘which test they took’, ‘which test they preferred’, ‘what is MAIN reason for [testing preference]’. Additionally, we asked research participants to provide reasons for said preferences and about their perception of the accuracy of the ‘new’ rapid test using a series of 4-point Likert scale questions.

### Statistical analysis

The principal outcome of interest was the choice of anti-HCV test: rapid or standard. Frequencies and measures of central tendency were used to describe sample characteristics and perception of HCV rapid testing (preferences, perceptions and reasons for testing choice). Fisher’s exact test was used to describe the associations between baseline characteristics and testing choice. Analyses were done using STATA 13.0 (StataCorp, College Station, Texas).

## Results

From June 2012 through August 2013, 129 participants were recruited, 127 (98.4%) completed survey questions about their point of care testing preference. A majority, 82.9% (n = 107) chose to receive the anti-HCV rapid test.

Table 1 describes testing choice by sample characteristics. The overall median age of participants was 25.1 years. More than half of participants were male (n = 88, 68.2%), white (n = 74, 57.4%), previously tested for HIV (n = 105, 82%), were self-reported HIV negative (n = 101, 78.3%), and were homeless (n = 101, 78.3). Most participants had previously accessed needle exchange in the previous 3 months (n = 95, 73.6%). The median years injecting was 4.2 (1.7-7.5). Testing preference did not significantly differ between any of the participant descriptors, HIV testing history, self-reported HIV status, service utilization or number of years injecting (Table 1).

**Table 1:** Characteristics of UFO study participants participating in the Rapid HCV testing study: (1) overall; and (2) in association with having chosen the rapid HCV test

**Characteristic**	**Overall (N = 129)**	**Rapid test (N = 107)**	**Standard blood draw (N = 22)**	**P-value**^**1**^
	**N**	**N (%)**	**N (%)**	
**Median age (range, IQR)[yrs]**	25 (23–27)	25 (23–27)	25 (23–27)	0.75
**Gender**				
Male	88	72 (81.8)	16 (18.2)	0.80
Female	40	34 (85.0)	6 (15.0)	
**Ethnicity**				
Non-white	55	48 (87.3)	7 (12.7)	0.35
White	74	59 (79.7)	15 (20.3)	
**Ethnicity**				
Asian, Asian-American	1	1 (100)	0 (0)	0.56
African American	5	4 (80.0)	1 (20.0)	
Filipino	1	1 (100)	0 (0)	
Latino/Hispanic	7	6 (85.7)	1 (14.3)	
Native American	5	3 (60.0)	2 (40.0)	
Caucasian	74	59 (79.7)	15 (20.3)	
Other	9	8 (88.9)	1 (11.1)	
Mixed	27	25 (92.6)	2 (7.4)	
**Previously tested for HIV**				
Yes	105	86 (81.9)	19 (18.1)	1.00
No	21	18 (85.7)	3 (14.3)	
Missing^*^	3		3	
**Self report anti-HIV results**				
Negative	101	83 (82.2)	18 (17.8)	1.00
Positive	5	4 (80.0)	1 (20.0)	
**Homeless, last 3 months**				
Yes	101	82 (81.2)	19 (18.8)	0.40
No	28	25 (89.3)	3 (10.7)	
**Completed grade 12**				
Yes	93	78 (83.9)	15 (16.1)	0.79
No	36	29 (80.6)	7 (19.4)	
**Seen health provider, last 3 months**				
Yes	64	52 (81.3)	12 (18.8)	0.82
No	64	54 (84.4)	10 (15.6)	
Missing^*^	1		1	
**Obtained new needles from NEP, last 3 months**				
Yes	95	77 (81.1)	18 (19)	0.43
No	33	29 (87.9)	4 (12.1)	
Missing^*^	1		1	
**Median years injecting (range, IQR)[yrs]**	4.2 (1.7-7.5)	4 (1.5-7.7)	4.9 (2.1-6.5)	0.51
**Inject every day in past 30 days**				
Yes	37	29 (78.4)	8 (21.6)	0.44
No	91	77 (84.6)	14 (15.4)	
Missing^*^	1		1	

*Missing values were not factored into the proportions or p-values presented.

^1^Fisher’s Exact Test for frequency and Kruskal-Wallis Test for median value comparison.

Table 2 presents perceptions of the ‘rapid test’ among the sample participants. Of those who chose the rapid test, 98% (105/107) responded to the questionnaire, and 81.9% (n = 86) of those preferred the rapid test to the phlebotomy-based standard test. More than half of participants who chose the rapid test did so because they wanted fast results (60.2%). Participants who chose the rapid test also reported feeling that the rapid test was just as or more accurate (62.3%), and the same or less painful (86.8%) than the conventional blood draw test. Most participants who received the rapid test agreed that they preferred receiving their results the same day (84.4%) and that they understood their results (97.5%). The majority of these participants would recommend the rapid test to a friend (93.9%). Very few participants agreed that it would have been better to wait a week to receive their results (3.1%). Perceptions of the rapid test among rapid testers did not differ by anti-HCV result (data not shown).

**Table 2:** Perceptions about HCV rapid test among young people who inject drugs in the UFO Study

**Variable**	**N (%)**
***Among all participants (N = 129)***	
**Belief in accuracy of rapid vs. Standard anti-HCV test**	
Much less accurate	3 (2.3)
Somewhat accurate	14 (10.9)
Just as accurate	70 (54.7)
More accurate	5 (3.9)
Much more accurate	3 (2.3)
Don’t know	33 (25.8)
***Among HCV Rapid Test takers (N = 107)***	
Main reason for choosing rapid anti-HCV test: (n = 83)*	
1. Wanted fast results	50 (60.2)
2. Rapid test is more convenient	10 (12.1)
3. I am afraid of needles or blood draws	1 (1.2)
4. I have bad veins	3 (3.6)
5. Rapid test is less painful	4 (4.8)
6. Rapid test requires less blood	6 (7.2)
7. Rapid test is less stressful	4 (4.8)
8. Rapid test is newer	1 (1.2)
9. I trust the research staff experience	2 (2.4)
10. I was concerned I wouldn’t get paid	2 (2.4)
**Compared to standard blood draw, getting a finger prick was: (n = 99)**	
Much more painful	4 (4.0)
More painful	9 (9.1)
About the same amount of pain	33 (33.3)
Less painful	29 (29.3)
Much less painful	24 (24.2)
**‘I have tested for HCV in the past and I prefer receiving my results the same day’ (n = 96)**	
Strongly agree	30 (31.3)
Agree	51 (53.1)
Disagree	13 (13.5)
Strongly disagree	2 (2.1)
**‘It would have been better to wait a week’ (n = 97)**	
Strongly agree	0 (0)
Agree	3 (3.1)
Disagree	66 (68.0)
Strongly disagree	28 (28.9)
**‘I understand the results of my test’ (n = 96)**	
Strongly agree	50 (52.1)
Agree	44 (45.8)
Disagree	1 (1)
Strongly disagree	1 (1)
**‘I would recommend rapid test to a friend’ (n = 98)**	
Strongly agree	42 (42.9)
Agree	50 (51)
Disagree	2 (2)
Strongly disagree	4 (4.1)

*Reasons for missing responses include interviewer error (n = 13) and a faulty skip pattern (corrected early in data collection) in which participants who preferred standard anti-HCV testing were not asked a follow-up question regarding testing preference reason (n = 7).

Of the 21 participants who opted for the standard HCV test and completed the questionnaire, 38.1% (n = 8) chose that test because they felt the test was older and more trusted. This was the most common reason given for choosing the standard test. Other participants stated that they did not want their results that day (n = 3, 14.3%), feeling that the standard was a more convenient test (n = 3, 14.3%), being afraid of fingers pricks (n = 2, 9.5%), and feeling that the standard test is less painful (n = 1, 4.8%). Overall, 17 (13.2%) of all participants felt that the rapid test was to some degree less accurate than the standard test “less quality control”, (n = 4), needs to be confirmed (n = 3), is too fast (n = 3), is too new (n = 3), or uses less blood (n = 2).

## Discussion

This study demonstrates that the OraQuick® anti-HCV rapid test by finger-prick is widely accepted and preferred in this sample of young adult PWID in San Francisco. This preference was not associated with sociodemographic characteristics, including age, gender, race/ethnicity, HCV and HIV testing history, or reported injection risk exposures. The principal reason participants indicated for preferring the rapid test was wanting fast results regarding anti-HCV status and with potentially less pain than with testing using a blood draw. An important indication that the test was acceptable was the widespread endorsement that they would recommend it to a friend. As hoped, these results suggest that rapid HCV testing has potential to increase HCV testing and knowledge of exposure to HCV infection. We also found that test preference did not differ by anti-HCV results; those testing positive were equally likely to favor the rapid test as those testing negative. This is important since follow-up testing for those testing anti-HCV positive will require a blood draw for HCV RNA testing. These results suggest that those at highest risk are not deterred from anti-HCV testing, or the potential for additional testing that may follow positive results.

In the U.S., there is growing concern about an emergent HCV epidemic in young adult PWID, not only in urban, but suburban and rural areas [13], and reflected by disproportionate increases in the number of acute HCV cases identified among young PWID and reported to the CDC in the past five years [8]. Our population is comparable to other studies of young adult PWID sampled in urban areas and in national surveys [17,25,53-56]. The large number of participants who were unstably housed or homeless presents another significant concern and potential opportunity for the implementation of this test. Evidence shows worrying rates of injection drug use among homeless youth [57]. These factors, and others, including mobility [58], coupled with barriers to phlebotomy-based testing faced by homeless youth [21,32,33], increase the imperative for more accessible options for HCV testing, prevention and linkages to care, and reinforce our focus on this population.

This test offers a critical opportunity to increase HCV testing among high risk young adult PWID, as well as access to other health services, including counseling to reduce risk behaviors and linkage to care. As evidenced by the introduction of rapid HIV testing, being able to offer rapid testing and immediate results may be the determining factor in increasing testing numbers, results disclosures and appropriate counseling services [43,45,59]. Comprehensive prevention programs that provide testing options, along with clean injection equipment, substance use treatment options, health education, and opportunities to access treatment and care, could potentially stem infection rates among young PWID [60,61]. Rapid HCV testing could be an essential component of such an inclusive approach. A majority (over 80%) of participants in this study report using needle exchange services. Implementation of rapid testing at such programs has potential to reach a large number of PWID. Knowing one’s HCV status is also critical to seeking and getting access to the appropriate care. Improved treatment options that are more tolerable and effective are likely to increase the demand for faster, more accessible testing options, together hopefully increasing access to care and treatment.

The rapid anti-HCV test remains limited by the need for nuanced counseling messages associated with anti-HCV testing, and by the need to confirm positive results. A single rapid test does not distinguish between those individuals with current HCV infection and those with past infection who have cleared the virus, nor will it give a clear picture of acute versus chronic infection. For this reason, viral RNA testing remains necessary for accurate knowledge of HCV infection status [5]. The HCV RNA test is associated with phlebotomy and lab work and participants must return for results, although use of dried-blood spots for HCV RNA testing is feasible and accurate [62,63] and testers might circumvent phlebotomy in some cases. In addition, testing sites without trained phlebotomists or the resources to offer RNA testing will have to provide referrals to clinical sites for people with positive HCV antibody. Many of the aforementioned barriers associated with testing that young PWID face, such as venipuncture and returning for results, thus remain a potential problem if someone tests anti-HCV positive.

Although the antibody testing does not give a clear picture of infection status or timeline, the rapid test may be more accessible and cheaper, facilitating serial testing among those who are negative and at high risk. Rapid testing could, then, help providers estimate a window of when someone newly acquires HCV, offering an important opportunity for prevention messages and referral to confirmatory testing and care, as new infections are marked by high viremia and these individuals are potentially at high risk of HCV transmission [60].

This study is limited by the fact that participants were already engaged in a study of HCV transmission and risk, had previously agreed to regular HCV testing, and may have received a HCV test prior to the baseline visit. Participants also understood that they would receive a phlebotomy blood draw as part of that study, regardless of whether they chose the rapid test or not. This study, therefore, does not necessarily represent individuals who are not already seeking and engaged in testing. Nevertheless, our population is highly reflective of the population at high risk of HCV, especially those in urban areas [13,16,57]. Generalizability of the favorable preference profile for the rapid test is also limited by the fact that all participants underwent phlebotomy-based testing for HCV RNA testing. Current HCV screening algorithms recommend reflexive testing for HCV viremia only among those testing anti-HCV positive [5]. Furthermore, as the rapid test may be implemented in settings where there are limited clinical staff, options for HCV RNA testing may be more limited. However, given the high rate of infection and the long seronegative viremic window observed in acute and early HCV infection, there is a need to find other methods to detect viremia in PWID in order to reduce the risk of HCV transmission during the seronegative window [51]. It is possible that our sample also had familiarity with rapid HIV testing, which could result in overestimates of the acceptability of HCV testing. We note, however, that HIV testing in the UFO Study is voluntary and not part of routine research testing. Since we have not measured the types of HIV testing that participants have had we cannot assess the effect of this potential exposure.

Through its accessibility in non-medical settings by lay-workers, by not initially requiring phlebotomy or accessible veins, and by providing immediate results, rapid testing offers an important opportunity to enhance prevention programs, and streamline access to care and treatment, adding a critical new dimension to preventing and treating HCV among vulnerable populations. Preference and acceptability should be assessed among those not already being tested, in real-world settings and at other community settings, such as substance abuse treatment centers, drop-in services, and needle exchange programs.

## Conclusion

Rapid anti-HCV antibody testing is likely to be a preferred and accepted method of testing among young adult PWID and has the potential to improve testing rates, enhance prevention programs, and increase access to care. Further study among other PWID groups and at other locations, especially in non-urban areas is needed.

## Competing interests

The authors declare they have to conflicts of interests.

## Authors’ contributions

BH contributed to study design, carried out participant surveys, analyzed and interpreted data, and drafted and revised manuscript. AB helped conceive of and coordinate the study, contributed to study design, managed data acquisition, helped interpret data and helped draft manuscript. AA assisted with design of the study survey, data collection and manuscript revisions. MY participated in data analysis and helped revise the manuscript. JE participate in concept of the study measures, data analyses and interpretation, and manuscript revisions. JH contributed to data interpretation, reviewing and revision of the final manuscript. KP conceived of the study, and participated in study design and coordination and reviewed and revised the manuscript. All authors read and approved the final manuscript.

## Previous presentation

Early versions of this paper were presented at the 2012 National Summit on HIV and Viral Hepatitis Diagnosis, Prevention and Access to Care, Washington, DC (November 2012) and at the UCSF Conference on Health Disparities, October 11, 2013.

## Pre-publication history

The pre-publication history for this paper can be accessed here:

http://www.biomedcentral.com/1471-2458/14/645/prepub
